# Are Morphometrics Sufficient for Estimating Age of Pre-Fledging Birds in the Field? A Test Using Common Terns (*Sterna hirundo)*

**DOI:** 10.1371/journal.pone.0111987

**Published:** 2014-11-06

**Authors:** Christy N. Wails, Stephen A. Oswald, Jennifer M. Arnold

**Affiliations:** Division of Science, The Pennsylvania State University, Berks Campus, Reading, Pennsylvania, United States of America; Institute of Ecology, Germany

## Abstract

Age is a key component of fitness, affecting survival and reproductive capacities. Where it is not possible to study known individuals from birth, morphometrics (predominantly patterns of plumage development for birds) are most often used to estimate age. Although criteria for age estimations exist for many species, the degree to which these criteria improve the precision of estimates remains to be tested, restricting their widespread acceptance. We develop a photographic tool for estimating ages of Common Tern (*Sterna hirundo*) chicks and test it using 100 human observers of varying prior experience across four breeding colonies (three North American sites and one European site) and under controlled laboratory conditions. We followed the design approach of other morphometric tools, expanding it to create a user-friendly guide (divided into six age groupings). The majority (86%) of observers improved in chick-aging accuracy when using the tool by an average of 20.1% (±1.4 SE) and correctly estimated 60.3% (±1.4) of chick ages. This was similar to the intrinsic aging ability of our best field observer (63.3%). Observers with limited experience showed the greatest increases in chick-aging accuracy over experienced observers who likely had established a method for estimating chick ages prior to using the tool. Even the best observers only correctly estimated ages of chicks 62.9% (±2.8) of the time in the field and 84.0% (±2.9) of the time in the lab when using the tool and typically underestimated ages. This indicates that developmental variation between individual chicks can prevent completely reliable age estimates and corroborates the few existing data that suggest that morphometric criteria fail to achieve robust levels of accuracy and may introduce error into studies that rely on them. We conclude that novel approaches for estimating age, not only morphometric criteria, must be pursued.

## Introduction

Age is a key component of fitness, often influencing survival and reproductive capacities [1]. Consequently, determining age is a key consideration in many areas of ecology, including population dynamics [2], life history evolution [3], development [4], senescence [5], behavioral ecology [6], and for conservation initiatives [7]. For birds, addressing population declines in a range of species requires detailed data on productivity that often rely on accurate estimations of chick ages [7], [8]. The nestling period can be studied more easily than other life stages [9] but, because chick survival is often strongly dependent on age, without an accurate way to estimate age, productivity can easily be over- or underestimated depending on survey frequency or methodology [10], [11].

While the most accurate way to determine age is to band chicks at hatching and visit nests at regular intervals [12], [13], this is time-consuming and labor-intensive, and often not practical due to difficulties accessing nesting sites [14], financial or logistical constraints [15], [16], or the sensitivity of species to human disturbance [17], [18]. Even colonial species often breed asynchronously, exhibiting a large spread around modal laying and hatching dates, e.g. [19], [20], and thus during a given study period there will be chicks of a wide variety of ages, e.g. [21], [22], further restricting the practicality of following many individual chicks from hatching.

As a result of these challenges, species-specific patterns of development have been identified for chicks and have been used to develop tools (such as age-predicting formulae, combinations of morphometric measurements, or visual guides) designed to assist in estimating ages of chicks (see Table S1 for a review). For many species, analyses of growth curves show that measurements of head-plus-bill length [23], culmen and tarsus length [24], primary feather length [25], wing length and body mass [26], and patterns of feather emergence and development [27], [28] are related to chick age. Age-predicting tools often employ various combinations of photographs or illustrations, descriptions of feather tract development, and skeletal measurements but most are either picture-based guides, that allow the user to estimate ages by comparing the chick to an image of a known-age chick, or published data tables or formulae that guide the user to estimate ages based on certain morphometric measurements (see Table S1 for details). Generally, species-specific tools designed for estimating ages of chicks are scarcer than publications elucidating or comparing age-related developmental changes that impact life history, breeding, or survivorship (Table S1).

Of those tools that do exist (Table S1), only three have provided some form of testing to indicate the accuracy that might result from their use [29]–[31]. However, these were not independent analyses of how well criteria improved age estimates of naïve observers but instead either a reassessment of a small number of chicks by a single experienced researcher [29] or differences between predictions from regression equations and known chick ages [30], [31]. Additionally, no previous studies have provided controlled tests that compared the accuracy of age estimates of observers before using a tool with the same observer using the tool, or included any assessment of the influence of prior experience or regional variation in bird morphology. Thus, robust tests are needed, not only to gauge the utility of these species-specific tools, but also to understand the accuracy of these and similar morphometric age estimation procedures routinely used in ecology.

In 2011 and 2012, we developed a tool to estimate ages of Common Tern (*Sterna hirundo*) chicks in the field following the same basic design as illustrated morphometric aging tools available for other species, e.g. [9], but expanded to a user-friendly, two-page photographic guide that can be printed double-sided for ease of use in the field (Fig. S1). This allowed users to quickly estimate ages of chicks without resorting to biometric measurements and regardless of previous experience. We then tested this tool in 2013 to evaluate its effectiveness at improving age estimates. We quantified its capacity to increase the ability of 100 investigators (of varying prior experience) to estimate chick ages accurately in trials at three field sites in North America, one in Europe, and under laboratory conditions. Specifically, we assessed the following predictions that visual tools based primarily on morphometrics can: (1) lead to improvements in age estimation for both inexperienced and experienced observers, (2) be used successfully at a range of different geographical locations, and (3) facilitate sufficiently high levels of precision in age estimations for these methods to be widely adopted.

## Methods and Experimental Design

### Ethics Statement

All activities were performed under appropriate permits (Canadian Wildlife Service Scientific Permits CA 0142, 0218, 0267, and 0308; Environment Canada Banding permits 10431V and 10431W; Ontario Parks Letter of Authorization to Conduct Research in a Provincial Park; and relevant permits held by collaborators at US and German sites) and approved by Pennsylvania State University’s Institute on Animal Care and Use Committee (protocols #28103 and #36295). Participants in laboratory tests provided written consent to participate in this study. For the field tests, written consent was provided electronically by the field site coordinators prior to tests and the volunteers present at the time of the testing visits provided additional verbal consent prior to actual tests. Pennsylvania State University’s Office of Research Protections determined that this research was of non-human/non-research status and thus further review by the Institutional Review Board or the Office for Research Protections was not required.

### Tool Development

Fieldwork was conducted at Gull Island, Presqu’ile Provincial Park, Ontario, Canada (43°59.1′N, 77°44.2′W) in the summers of 2011 and 2012, although additional data collected at the same site from 2008–2010 was used in biometric summaries in the tool. Each year, nests were marked with numbered stakes as Common Terns initiated clutches and subsequently monitored on a near-daily basis to ensure accurate determination of hatching dates. Individual chicks were banded at hatching. Chicks were hand-caught, photographed, and measured (mass, head-plus-bill length, and wing length) regularly (every 1–7 days) from hatching to fledging. Chicks were selected arbitrarily from those available as these were years of intense nest predation by Black-crowned Night Herons (*Nycticorax nycticorax*). Therefore, since sample sizes of photographs and head-plus-bill measurements among the very oldest chicks were low, we supplemented these data with corresponding measurements of Common Tern chicks at other North American sites (see Fig. S1 for details). While photographing, chicks were placed on a small white table with a stationary ruler to establish a uniform photograph background and scale. An Olympus SP-590UZ camera (Olympus America Inc., 3500 Corporate Parkway, Center Valley, PA 18034) was mounted so the lens was approximately 35 cm above the platform. Photographs of the whole body of the chick and a detailed photograph of the outstretched wing were taken.

**Figure 1:**
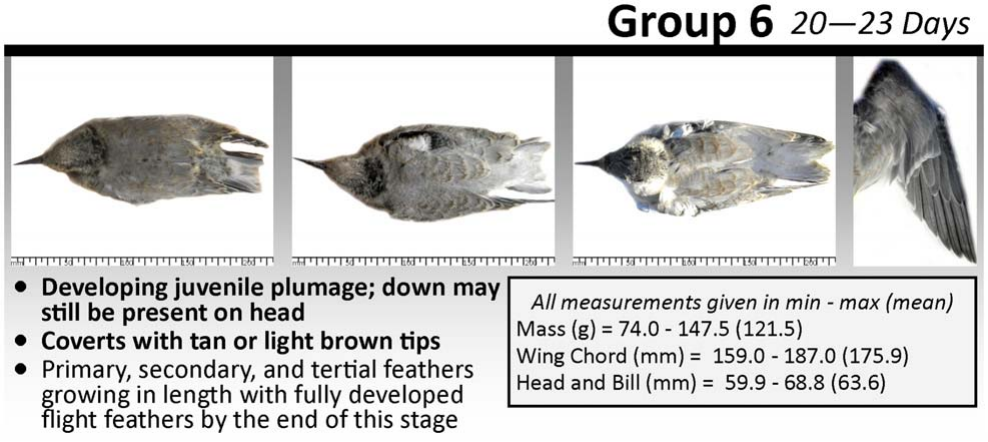
Example of age group from the tool. Age group 6 (chicks 20–23 days old) is shown (full tool is provided as Fig. S1). Within the age group, pictured chicks increase in age from left to right; an image of the outstretched wing of the oldest chick is provided. Morphometric data summaries and key diagnostic characteristics are also shown.

The tool is a double-sided photographic card (Fig. S1) designed for use in the field and was prepared in Microsoft Publisher 2010 (version 14.0; Microsoft, 15010 NE 36th Street, Redmond, WA 98052). Chicks were divided into six distinct age groups (to facilitate ease of use in the field) based primarily on feather development and commonly identified growth phases, e.g. [22], approximately 3–5 days in length (0–3 days, 4–7 days, 8–12 days, 13–15 days, 16–19 days, 20–23 days; Fig. 1). Of the 73 chicks available to be photographed at Presqu’ile, the final photographs used were selected to demonstrate the variation of color and pattern differentiation that can occur within each specific age range. Raw photographs were selected based on clarity and uniformity. Photographs were then scaled identically within the age group in Adobe Photoshop (CS5 and CS6; Adobe Systems Inc., 345 Park Avenue, San Jose, CA 95110) using the rulers in images, and a white background and clear scale were superimposed. Each chick was pictured only once on the tool with the exception of two individuals pictured in separate age groups due to the limited number of usable photographs available. Biometric summaries (means and ranges) were calculated for measurements of chicks in each age group (sample size range per age group: mass, 270–1581; head-plus-bill length, 7–96; wing length, 13–57). Biometrics were chosen based on those regularly used to describe chick development in this species (Table S1) [21].

### Testing Approaches

We used three different testing approaches in 2013. In each, human test subjects (“observers”, n = 100) estimated ages of Common Tern chicks over a number of trials held on the same day to address our predictions of the effects of the tool on age estimation:

1. *Learning-phase testing* (Gull Island, Presqu’ile, ON and Little Island, Oneida Lake, NY [43°14.2′N, 76°0.0′W]) was used to quantify the effects of tool use and learning among 11 observers. An observer estimated ages of chicks over five successive trials: three times initially without using the tool and twice subsequently with the tool.
2. *Direct testing* (Bird Island, MA [41°40.2′N, 70°43.0′W] and Banter See, Wilhelmshaven, Germany [53°30.7′N, 08°06.3′E]) was used when learning-phase testing was not possible. We quantified the *immediate* effect of using the tool by having each of the two observers estimate ages of chicks twice: first without the tool and then with the tool.
3. *Laboratory testing* (at Pennsylvania State University, Berks Campus, Reading, PA), using whole-body photographs of chicks, was performed to facilitate a large sample size (87 observers) that included a range of less-experienced observers. This approach only differed from direct testing in that the image of the chick appeared on an overhead projector. An outstretch wing and ruler (for scale) were visible in all images.

All observers estimated ages of all chicks in each trial (these were known-age [banded within 48 hours of hatching in most cases] but ages were never disclosed to observers until after all trials). Chicks that were not banded on their hatch date were aged using egg signs from previous visits (e.g. “pipped” or “starred” eggs), information on siblings, and whether or not their plumage was still damp from hatching. In all field tests, Common Tern chicks were held in a variety of temporary collection boxes (e.g. car dboard boxes, plastic crates) with an assistant removing chicks individually from the box in an arbitrary sequence and recording bands to identify the chicks. Each observer then recorded their estimates of chick ages either with or without access to the tool (according to the trial).

All observers were instructed to use visual cues as primary diagnostics rather than take time to procure detailed measurements because age estimates were strictly limited to 30s per chick (as necessary for time-constraints of most field situations). After each trial, laboratory observers recorded the main features they used in their determination of chick ages. In all trials, except those at Little Island, the same chicks (or images) were used in each subsequent trial, just presented in different orders.

### Analysis

We analyzed results from the three testing procedures separately, as a result of important differences in methodologies. Before analyzing learning-phase data using an information-theoretic approach [32], we tested the key assumption that learning did not take place for observers during repeated trials when not using the tool. We used a Wilcoxon Signed Rank test to compare the percent of chicks correctly aged for each of eight observers between trial 1 and trial 3 (i.e. first time estimating age and third time estimating age in repeated trials without the tool). Three observers were omitted from this prior analysis because several chicks had to be excluded as a result of inconsistencies in their recorded hatch date which resulted in reduced sample sizes for these individuals in the first trial only.

Chick-aging accuracy of observers (correct or incorrect estimate of the age of each chick) was analyzed in generalized linear mixed models (GLMMs) with binomial errors and logit links, for both learning-phase data and laboratory data separately. This approach has been used previously for analyzing repeated measures treatments in presence of covariates [33]. Use of tool or not (“Tool”), number of previous trials of the same type (either with or without the tool, [“Learning”], for learning-phase testing only), field experience (≥1 yr field experience with Common Terns = “Experienced” [n = 6] in field tests; any previous experience with birds = “Experienced” [n = 28] in laboratory testing, [“Exp”]), chick age group (2–6 for field data as there were no Group 1 chicks in tests at Gull Island; 1–6 for laboratory data, [“Group”]), colony site location ([“Colony”], for learning-phase testing only), and date ([“Date”], for laboratory testing only) were entered as fixed factors. Observer and chick identities were included as random factors. For each dataset, the maximal model was constructed (including all covariates and all two-way and three-way interactions that were biologically-meaningful). Model reduction using AIC_c_ model selection [32] was used to identify the most parsimonious model using the R package *MuMIn* [34]. Evidence ratios [35] were used to compare the best model with the highest-ranking competing model that did not include tool use as a predictor. Where top models (ΔAIC_c_<2) differed in their inclusion of tool use as a predictor, we used model averaging across all models [36] to determine the relative importance of tool use compared to other predictor variables. Statistical comparison of direct testing sites was not possible due to limited numbers of observers.

We summarized the key identification features used by observers in each trial. Means are presented with ± SE and medians with [lower quartile, upper quartile] unless otherwise stated.

## Results

**Figure 2:**
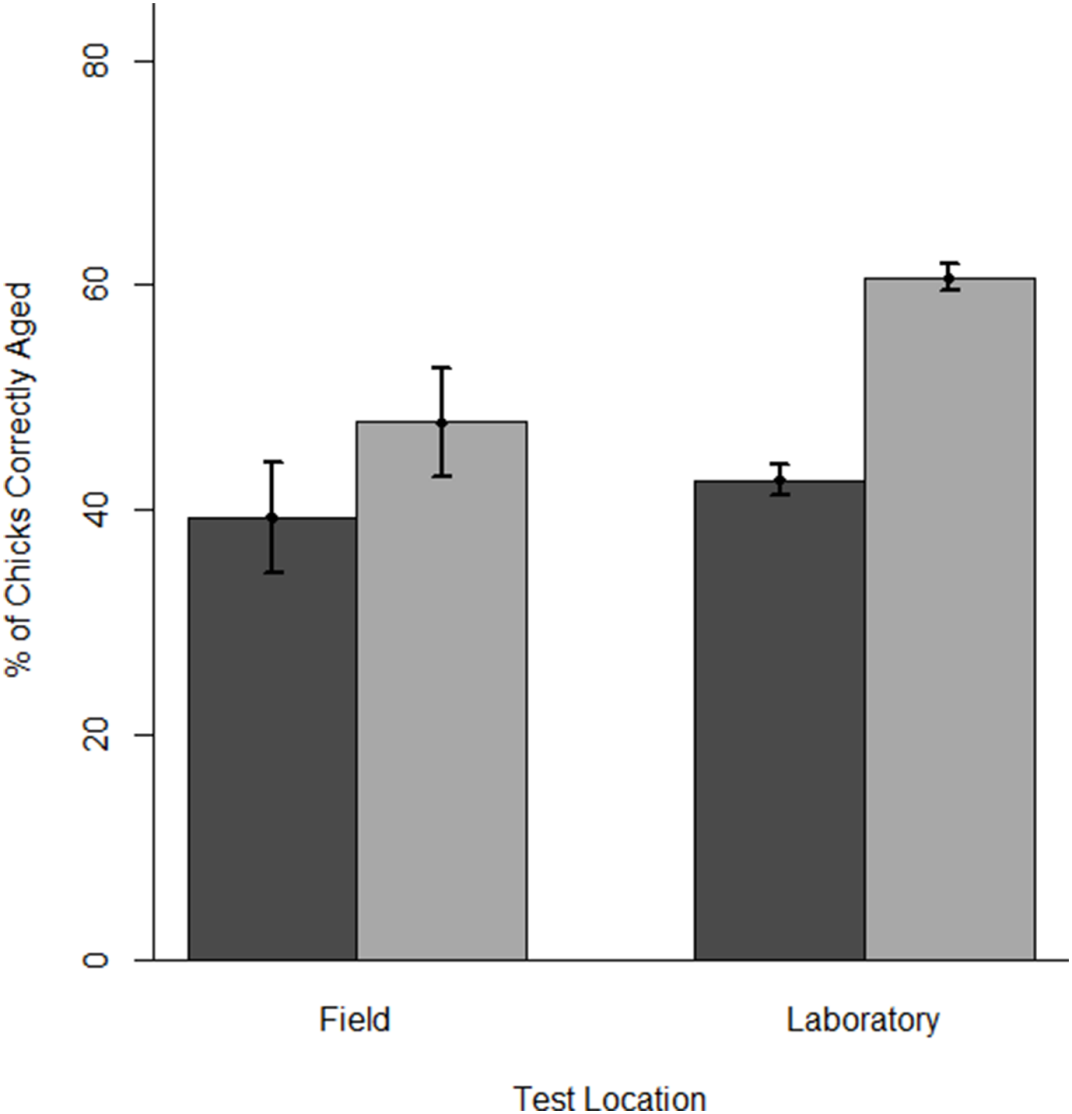
Mean chick-aging accuracy without and with the tool for observers in field and laboratory tests. Chick-aging accuracy is the percentage of chicks aged correctly by observers when not using (dark gray) and when using (light gray) and the aging tool. Error bars are ±1 SE.

Overall, the tool improved chick-aging accuracy by 16.8% (±1.5), with 86% of observers showing improvement (on average: 20.1±1.4%) and only 14 not improving (range: −10%–0%). Observers in laboratory tests showed a greater improvement in chick-aging accuracy than observers in field tests (18.0±1.6% vs. 8.4±2.6% respectively, Fig. 2). When using the tool, the best five observers from laboratory tests and field tests (those achieving the greatest chick-aging accuracy) correctly estimated 84.0% (±2.9) of chick ages and 62.9% (±2.8) of chick ages, respectively. In the field, the tool improved chick-aging accuracy at all sites (Bird Island: 15.9%, Banter See: 6.7%, Gull Island: 9.2±6.3%, Little Island 6.7±2.9%).

Eighty-four percent of laboratory observers mostly used characteristics of feather development to estimating chick ages without the tool and this increased to 95% when using the tool (Table 1). Observers in field and laboratory tests had the most difficulty in estimating ages of chicks in later age groups (Groups 4–6; 13–23 days of age) both with and without the tool (Table S2) but underestimated age for Groups 5 and 6 (16–23 days of age) more when using the tool. Additionally, observers late in the season at Gull Island and Little Island frequently underestimated ages of chicks in Group 2 (4–7 days of age) when using the tool.

**Table 1:** Morphometric features most often used for aging estimates prior to and when using the tool.

Before Tool				
Feathers on Wing	Feathers on Body	Sizing Ruler on Pictures		Egg Tooth Presence
44.8%	39.1%	16.1%		0.0%
**After tool**				
**Feathers on Wing**	**Feathers on Body**	**Descriptions of Feathers**	**Sizing Ruler on Pictures**	**Egg Tooth Presence**
57.5%	23.0%	14.9%	2.3%	2.3%

Data are percents of responses of observers in laboratory tests (n = 87) when asked which criteria they used to estimate age.

### Field Tests

When not using the tool, there was no evidence for learning (any improvement in observer ability to age Common Tern chicks during learning-phase trials) during consecutive trials (Wilcoxon Signed Rank W_8_ = 17.5, p = 0.612). Even though our sample of observers in this test was small (only eight were available across all five trials), performance decreased slightly which is the opposite of what would be expected if observers were learning (1^st^ vs. 3^rd^ trial without tool: 50.0 [38.8, 60.9] vs. 45.0 [38.8, 50.0]). Additionally, changes in aging accuracy between consecutive trials (either both with the card or both without the card) were not retained in the best GLMM model for learning-phase testing (Table 2). For both direct testing and learning-phase field tests, observers did show marked improvement in chick-aging accuracy when using the tool (Fig. 2, Table 2). This improvement was retained in the best GLMM model for learning-phase over the second-best competing model (without tool use; Evidence Ratio = 1.24, Table 2). Model averaging of coefficients across all GLMMs indicated that the age of chicks was the most important determinant of observer aging performance (Relative Importance = 0.98), followed by prior experience (0.67), colony location (0.64) and tool use (0.62, Table S3).

**Table 2:** Highest-ranked GLMMs (ΔAIC_c_<2) for observer chick-aging accuracy (correct or incorrect estimation of chick age) in learning-phase trials.

ModelRank	Model	k	NegativeLogLikelihood	AIC_c_	Δ AIC_c_	AIC_c_Weight
1	Exp+Tool+Group+Colony+Exp: Colony	10	−298.455	617.4	0.00	0.118
2	Exp+Group+Colony+Exp: Colony	9	−299.719	617.8	0.44	0.095
3	Tool+Group	7	−301.967	618.2	0.78	0.080
4	Group	6	−303.048	618.3	0.88	0.076
5	Exp+Tool+Group+Colony+Exp:Tool+Exp: Colony	11	−298.219	619.0	1.63	0.052
6	Exp+Tool+Group+Colony+Learning+Exp:Colony	11	−298.300	619.2	1.79	0.048

Fixed factors included tool use (“Tool”), number of previous trials of the same type (either with or without the tool [“Learning”]), field experience (≥1 yr field experience with Common Terns = experienced, [“Exp”]), chick age group (2–6 as there were no Group 1 chicks in tests at Gull Island [“Group”]), and colony site location (“Colony”). Tool use was retained in 67% of the top six ranked models: models ranked second and fourth were the only ones not to retain tool use as a factor. Model selection (reduction) began from the maximal model (not shown) that included all two-way and three-way interactions. Number of parameters (k), and AIC_c_ weights are given.

**Figure 3:**
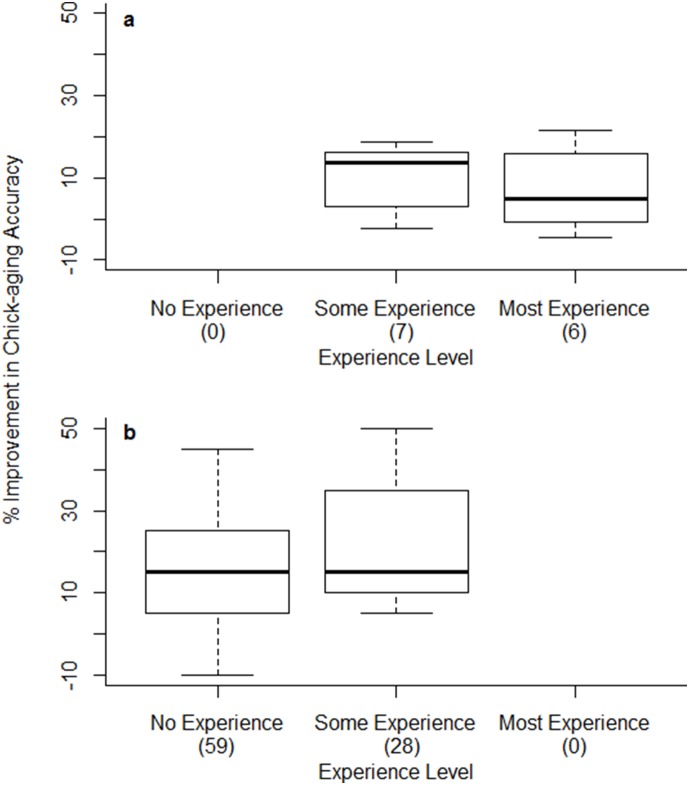
Boxplots of improvement in chick-aging accuracy when using tool for observers of different prior experience in (a) field and (b) laboratory tests. Improvement in chick-aging accuracy is the difference in the percentage of chicks aged correctly when using the tool versus without the tool. Sample sizes of observers are given in parentheses next to axis labels. “No Experience” = no prior experience working with any birds, “Some Experience” = <1 year working with birds (including Common Terns) [for laboratory trials this was any previous experience with birds], and “Most Experience” = 1+ years working with Common Terns.

Most field observers improved (by 1.4%–21.7%); only four did not (−4.5%–−0.8%). Observers with some experience (those with <1 year of experience working with Common Terns) showed a greater level of improvement than more experienced personnel (Table 2, Fig. 3).

### Laboratory Trials

Eighty-eight percent of observers in laboratory tests showed improvement (by 5%–54.58%) when using the tool over when not doing so; only ten (11.5%) did not improve (−10%–0%). Use of the tool was retained in the most parsimonious GLMM (Table 3) and was strongly supported in comparison to the highest-ranking competing model without tool use as a predictor (model rank 17, Table 3; Evidence Ratio = 4.4×10^26^), however, there was no evidence for any effect of prior experience on ability to age chicks in the laboratory (experience was not retained in the best model (Table 3, Fig. 3).

**Table 3:** Highest-ranked (ΔAIC_c_<2) and other GLMMs for observer chick-aging accuracy accuracy (correct or incorrect estimation of chick age) in laboratory trials.

ModelRank	Model	k	Negative LogLikelihood	AIC_c_	Δ AIC_c_	AIC_c_Weight
1	Group+Tool	9	2147.0	4312.0	0.00	0.293
2	Exp+Group+Tool+Exp: Tool	11	2145.5	4313.0	1.06	0.173
3	Date+Group+Tool	15	2141.6	4313.3	1.28	0.155
4	Exp+Group+Tool	10	2146.6	4313.3	1.28	0.154
17	Group	8	2209.3	4434.7	122.67	6.75×10^−28^
18	Date+Group	14	2203.9	4435.9	123.92	3.61×10^−28^

Fixed factors included tool use (“Tool”), experience (any previous experience with birds = experienced, [“Exp”]), chick age group (“Group”), and test date (“Date”). Tool use was retained in 89% of the top 18 ranked models: models ranked 17 and 18 were the only ones not to retain tool use as a factor. Model selection (reduction) began from the maximal model (not shown) that included all two-way and three-way interactions. Two models which did not include tool use as a predictor are shown for comparison. Number of parameters (k), and AIC_c_ weights are given.

## Discussion

We developed a photographic field tool designed to improve estimates of age of Common Tern chicks for users of varying levels of experience (Fig. 1, Fig. S1). More importantly, we provided an extensive, independent validation of the tool, showing that it improved the accuracy of age estimations for 86% percent of our 100 observers, by 20% on average. This level of improvement is similar to the difference in intrinsic ability (without the tool) between a naïve observer and our best field observer, who had over a decade of experience working with Common Terns. The tool was universally effective, improving estimates for both experienced and inexperienced observers across sites in North America and Europe. Unsurprisingly, observers with a little (<1 year) or no experience showed the greatest increases in chick-aging accuracy (Fig. 3), presumably because more experienced observers had already established a method for aging chicks prior to using the tool. This also explains why observers in laboratory tests (with little prior experience) showed more marked improvement with the tool than the more experienced field observers (18% vs. 8% improvement on average, Fig. 3).

Despite the obvious improvements when using the tool, even the best five field observers (those achieving the greatest chick-aging accuracy) only correctly estimated the chick age groups 63% of the time, suggesting that developmental variation between individual chicks appear to hinder completely reliable age estimates. Gender, parental quality, and hatching date and order are known factors that influence growth and survival of tern chicks [20]–[22] and any of these may have been responsible for the observed developmental variation. Chicks between 13–19 days of age (Groups 4 and 5) were the most challenging for age estimation both with and without the tool (Table S2). This may be because they lacked the clear signs of the oldest age group (fledging-age chicks that lacked down on their heads and wings and had well-developed primaries and head caps, Fig. 1) but had had many days since hatching to diverge in their individual rates of development. Additionally, when using the tool, observers tended to underestimate the age of the oldest chicks (Groups 5 and 6; 16–23 days) more frequently than without it (Table S2). In a study using molt patterns, Parr [19] noted that when using his aging criteria (molt and development of primary feathers) he consistently underestimated ages of older Red Grouse (*Lagopus lagopus*) chicks that exhibited slow development (‘runts’). Presumably, by providing a consistent frame of reference, both our tool and that of Parr [19] increase the possibility of underestimating age for any older chicks that have less well-developed plumages.

Our tool has a wide range of features that can be used to estimate age but most observers cited feather development as the main feature they used for age estimation. We believe this focus on feather development was a main reason our inexperienced observers improved so quickly, as other morphometrics and visual cues are generally more variable, but it may also have led to the consistent trend of underestimating ages for late-hatched chicks. Poor dietary conditions can retard mass growth and primary feather development disproportionately [20], [37], [38] leading to underestimations in age if using only feather development [31]. At Gull Island and Little Island, our observers experienced difficulty estimating ages of young chicks (Group 2; 4–7 days) late in the breeding season. This was probably because late-hatched chicks are generally offspring of young adults or re-nesting birds [37], which commonly exhibit retarded provisioning, growth, and development [22], [39].

Previous studies have suggested, but not shown, that using a combination of morphometric measurements is a practical way for estimating age (see Table S1). However, only three of these studies developed tools based on morphometrics *and* provided some estimate of their effectiveness, either a single observer estimating ages for very few chicks [29] or statistical estimates of the fit of predictive regression equations [30], [31]. Although we show through an extensive testing protocol that visual aging tools based in morphometrics do improve accuracy, this leads at best to only 63% chick-aging accuracy in the field and 84% chick-aging accuracy in the laboratory (performance of top five observers in both tests). While promoting the use of feather development cues from photographs instead of purely biometric measurements facilitated rapid age estimations in our study, it could be argued that quantitative measurements may provide higher levels of accuracy. However, the ability of our best observers in our study to estimate chick ages correctly 60–80% of the time is consistent with conclusions for other species from less extensive tests of tools based more on quantitative measurements, e.g. [29], [31]. Thus, estimating ages using guides based on morphometric criteria alone may be unreliable and lead to a high level of error in age estimation. Even within the same year and under similar environmental conditions, individual chicks can experience variations in growth and development [21], [40], [41] which can cause bias in aging estimates from standardized morphometric criteria. Therefore, studies that rely on estimating age solely from morphometrics may be subject to inaccuracies that need to be accounted for during experimental design.

For conservation initiatives that require robust estimates of reproductive success and are often based on criteria using chick age, accommodating age-specific chick survival using age estimates from morphometric tools may misrepresent individual chick survival and bias productivity estimates. The development of species-specific tools for estimating age should therefore focus on using innovative approaches rather than relying solely on morphometrics (Table S1). Seasonality, diet, parental quality, and environmental factors all influence growth and development [21], [40], [41] and alternative approaches should seek to unite this information with visible morphometrics.

Despite the possible short-comings we highlight, morphometric tools for estimating age in the field are currently the best solution to increase accuracy of age determination for birds of unknown hatch-date in field studies. Such conditions commonly arise where regular nest visits are impractical, for example for reasons of disturbance [18], extensive study area, e.g. [42] or other logistical limitations [15], [16]. Therefore, we still recommend the widespread use of existing species-specific aging tools following their extensive testing but suggest that new tools should use more than just morphometric characteristics. One promising approach would be to combine metadata on important variables that affect growth and development (e.g. seasonal timing, dietary status, parental quality) with morphometric characteristics to accommodate problematic individual variation in growth rate.

## Supporting Information

Figure S1 **Morphometric tool for estimating ages of Common Tern chicks in the field.**
(DOCX)

Table S1 **Review of published species-specific aging criteria.**
(DOCX)

Table S2 **Bias in estimation errors during all tests of the tool.**
(DOCX)

Table S3 **Model averaging results for learning-phase tests.**
(DOCX)

File S1 **Biometric measurements used in tool development.**
(XLSX)

File S2 **Data from all testing procedures.**
(XLSX)
