# Supplementary material for: Are Morphometrics Sufficient for Estimating Age of Pre-Fledging Birds in the Field? A Test Using Common Terns (Sterna hirundo)
Source: PLoS One. 2014 Nov 6;9(11):e111987. doi: 10.1371/journal.pone.0111987 (PMC4222966; doi:10.1371/journal.pone.0111987)

**Fig. S1.** The *Common Tern Chick Aging Guide* - a 8.5 x 11” visual tool for estimating ages of Common Tern chicks in the field. The first image depicts the ‘front’ side of the tool, featuring younger chicks from 0 – 12 days of age. The second image depicts the ‘back’ side of the tool, featuring older chicks from 13 – 23 days of age. The tool indicates the age group and the left-most photograph of the group represents the youngest individual within the age range and age progresses to the right. A clear image of the wing of the oldest age chick within the group is also provided. Morphometric data recorded in the field from 2008 – 2013 were summarized on the tool and key diagnostic points provided for each age group are presented.


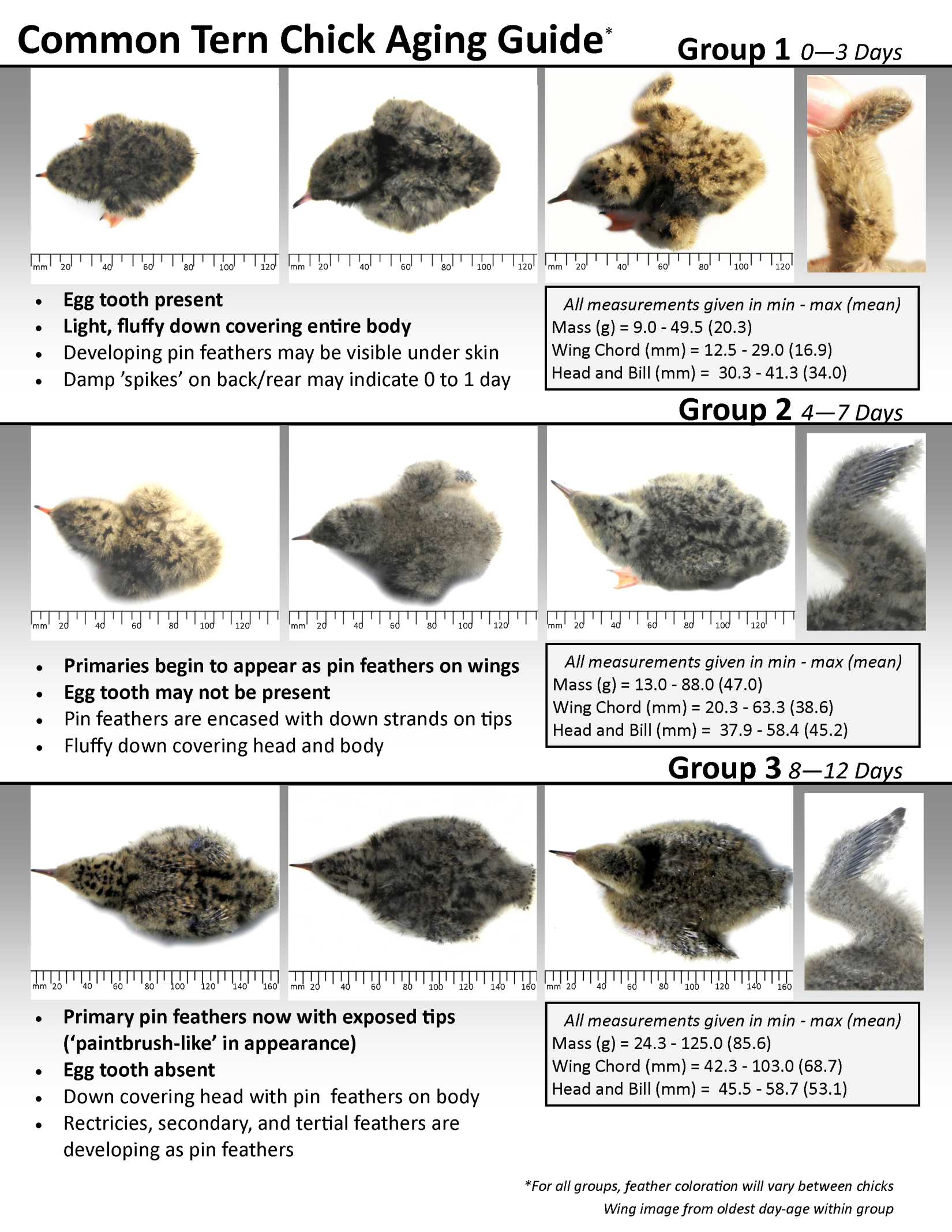


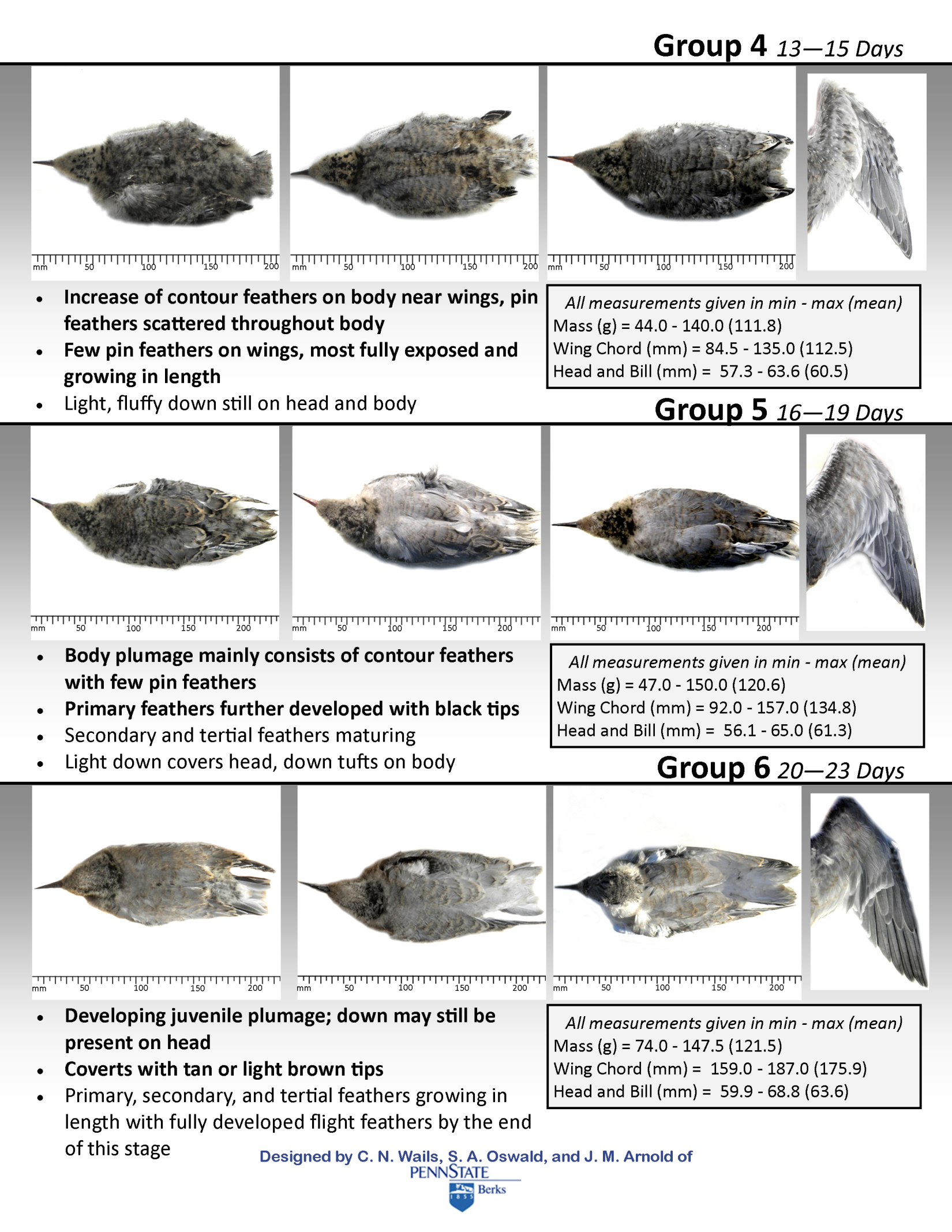

Supplement: Figure S1 — Morphometric tool for estimating ages of Common Tern chicks in the field. (DOCX) [file pone.0111987.s001.docx]
