# Supplementary material for: Are Morphometrics Sufficient for Estimating Age of Pre-Fledging Birds in the Field? A Test Using Common Terns (Sterna hirundo)
Source: PLoS One. 2014 Nov 6;9(11):e111987. doi: 10.1371/journal.pone.0111987 (PMC4222966; doi:10.1371/journal.pone.0111987)
Supplement: Table S1 — Review of published species-specific aging criteria. (DOCX) [file pone.0111987.s002.docx]

**Table S1.** Review of published species-specific aging criteria.

| **Species** | **Aging Method^1^** | **BB^2^** | **GS^3^** | **CD^4^** | **Reference** |
| --- | --- | --- | --- | --- | --- |
| American Goldfinch (*Carduelis tristis*) | Plumage descriptions, mass, skeletal and feather measurements |  |  |  | [1] |
| Atlantic Puffin (*Fratercula arctica*) | Mass, skeletal measurements |  |  |  | [2] |
| Barn Swallows (*Hirundo rustica*) | Plumage descriptions, mass, feather measurements |  |  |  | [3] |
| Black Vulture (*Coragyps atratus*) | Mass, skeletal and feather measurements |  |  |  | [4] |
| Black-crowned Night Heron (*Nycticorax nycticorax*) | Mass, skeletal measurements |  |  |  | [5] |
| Blue-winged Teal (*Anas discors*) | Plumage descriptions, feather measurements, bill spots |  |  |  | [6] |
| Bonelli's Eagle (*Hieraaetus fasciatus*) | Mass, skeletal and feather measurements |  |  |  | [7] |
| Broad-winged Hawk (*Buteo platypterus*) | Skeletal and feather measurements |  |  |  | [8] |
| California Gull (*Larus californicus*) | Plumage descriptions, mass, skeletal and feather measurements |  |  |  | [9] |
| Canada Goose (*Branta canadensis moffitti*) | Plumage descriptions |  |  |  | [10] |
| Canvasback (*Aythya valisineria*) | Plumage descriptions, mass, skeletal and feather measurements |  |  |  | [11] |
| Carolina Wren (*Thryothorus ludovicianus*) | Plumage descriptions, mass, skeletal and feather measurements |  |  |  | [1] |
| Chestnut-collared Longspur (*Calcarius ornatus*) | Plumage descriptions, mass, skeletal and feather measurements |  |  |  | [1] |
| Common Grackle (*Quiscalus quiscula*) | Mass, skeletal and feather measurements |  |  |  | [12] |
| Common Tern (*Sterna hirundo*) | Plumage descriptions, mass, skeletal and feather measurements |  |  |  | [13] |
| Dusky Flycatcher (*Empidonax oberholseri*) | Plumage descriptions, mass, skeletal and feather measurements |  |  |  | [1] |
| Eagle Owl (*Bubo bubo*) | Plumage descriptions, mass, skeletal measurements |  |  |  | [14] |
| Eastern Kingbird (*Tyrannus tyrannus*) | Plumage descriptions, mass, skeletal and feather measurements |  |  |  | [15] |
| Eastern Phoebe (*Sayornis phoebe*) | Plumage descriptions, mass, skeletal and feather measurements |  |  |  | [15] |
| Ferruginous Hawk (*Buteo regalis*) | Plumage descriptions, body length |  |  |  | [16] |
| Great Egret (*Casmerodius albus*) | Mass, skeletal measurements |  |  |  | [5] |
| Gyrfalcons (*Falco rusticolus*) | Mass, feather measurements |  |  |  | [17] |
| Hawaiian Goose (*Branta sandvicensis*) | Plumage descriptions |  |  |  | [18] |
| Hawaiian Stilt (*Himantopus mexicanus knudseni*) | Plumage descriptions, mass, skeletal and feather measurements |  |  |  | [19] |
| Herring Gull (*Larus argentatus*) | Skeletal measurements |  |  |  | [20] |
| Herring Gull (*Larus argentatus*) | Skeletal measurements |  |  |  | [21] |
| Indian Eagle-owl (*Bubo bengalensis*) | Plumage descriptions, mass, skeletal measurements |  |  |  | [22] |
| Laughing Gull (*Larus atricilla*) | Skeletal measurement |  |  |  | [23] |
| Laughing Gull (*Larus atricilla*) | Skeletal measurement |  |  |  | [24] |
| Mockingbird (*Mimus polyglottos*) | Plumage descriptions |  |  |  | [25] |
| Northern Harrier (*Circus cyaneus*) | Mass, skeletal and feather measurements |  |  |  | [26] |
| Prairie Falcon (*Falco mexicanus*) | Plumage descriptions, body length |  |  |  | [27] |
| Prothonotary Warbler (*Protonotaria citrea*) | Plumage descriptions, mass, skeletal and feather measurements |  |  |  | [28] |
| Red Grouse (*Lagopus lagopus*) | Plumage descriptions |  |  |  | [29] |
| Red-shouldered Hawk (*Buteo lineatus*) | Mass, skeletal and feather measurements |  |  |  | [30] |
| Red-tailed Hawk (*Buteo jamaicensis*) | Plumage descriptions, body length |  |  |  | [31] |
| Red-winged Blackbird (*Agelaius phoeniceus*) | Plumage descriptions, mass, skeletal and feather measurements |  |  |  | [32] |
| Snowy Egret (*Egretta thula*) | Mass, skeletal measurements |  |  |  | [5] |
| Song Sparrow  (*Melospiza melodia*) | Plumage descriptions, mass, skeletal and feather measurements |  |  |  | [1] |
| Sprague's Pipit (*Anthus spragueii*) | Plumage descriptions, mass, skeletal and feather measurements |  |  |  | [1] |
| Surf Scoter (*Melanitta perspicillata*) | Plumage descriptions, mass, skeletal and feather measurements |  |  |  | [33] |
| Turkey Vulture (*Cathartes aura*) | Mass, skeletal and feather measurements |  |  |  | [4] |
| Ural Owl (*Strix uralensis*) | Mass, skeletal and feather measurements |  |  |  | [34] |
| Wrentit (*Chamaea fasciata*) | Plumage descriptions, mass, skeletal and feather measurements |  |  |  | [1] |
| Yellow-headed Blackbird (*Xanthocephalus xanthocephalus*) |  | X |  |  | [35] |
| House Sparrow (*Passer domesticus*) |  | X |  |  | [36] |
| Northern Pileated Woodpecker (*Ceophloeus pileatus abieticola*) |  | X |  |  | [37] |
| Pinyon Jay (*Gymnorhinus cyanocephalus*) |  | X |  |  | [38] |
| Horned Lark (*Eremophila alpestris*) |  | X |  |  | [39] |
| Double-crested Cormorant (*Phalacrocorax auritus*) |  | X |  |  | [40] |
| Downy Woodpecker (*Picoides pubescens*) |  | X |  |  | [41] |
| Wood Duck (*Aix sponsa*) |  | X |  |  | [42] |
| Eurasian Sparrowhawk (*Accipiter nisus*) |  | X |  |  | [43] |
| Savannah Sparrow (*Passerculus sandwichensis*) |  | X |  |  | [44] |
| White-winged Scoter (*Melanitta deglandi*) |  | X |  |  | [45] |
| Cackling Canada Goose (*Branta canadensis minima*) |  | X |  |  | [46] |
| Common Sandpiper (*Actitis hypoleucos*) |  | X |  |  | [47] |
| Common Blackbird (*Turdus merula*) |  | X |  |  | [48] |
| Great Black-backed Gull (*Larus marinus*) |  | X |  |  | [49] |
| Indian Barn-Owl (*Tyto alba stertens*) |  | X |  |  | [50] |
| Long-eared Owl (*Asio otus*) |  | X |  |  | [51] |
| White-crowned Sparrow (*Zonotrichia leucophrys*) |  |  | X |  | [52] |
| Northern Harrier (*Circus cyaneus*) |  |  | X |  | [53] |
| Black-legged Kittiwake (*Rissa tridactyla*) |  |  | X |  | [54] |
| Herring Gull (*Larus argentatus*) |  |  | X |  | [55] |
| Purplish-backed Jay (*Cyanocorax beecheii*) |  |  | X |  | [56] |
| Bachman's Sparrow (*Peucaea aestivalis*) |  |  | X |  | [57] |
| Greater Snow Goose (*Chen caerulescens atlanticus*) |  |  | X |  | [58] |
| Tree Swallow (*Tachycineta bicolor*) |  |  | X |  | [59] |
| Northern Pintail (*Anas acuta*) |  |  | X |  | [60] |
| Great Tit (*Parus major*) |  |  | X |  | [61] |
| Eurasian Blue Tit (*Parus caeruleus*) |  |  | X |  | [61] |
| Common Tern (*Sterna hirundo*) |  |  | X |  | [62] |
| Common Murre (*Uria aalge*) |  |  | X |  | [63] |
| Spotted Owlet (*Athene brama*) |  |  | X |  | [64] |
| Tufted Duck (*Aythya fuligula*) |  |  |  | X | [65] |
| Brewer's Blackbird (*Euphagus cyanocephalus*) |  |  |  | X | [66] |
| Eastern Bluebird (*Sialia sialis*) |  |  |  | X | [67] |
| Sooty Tern (*Onychoprion fuscatus*) |  |  |  | X | [68] |
| Common Tern (*Sterna hirundo*) |  |  |  | X | [68] |

^1^Articles outlining characteristics and methods to estimate ages of chicks.

^2^Articles outlining breeding biology of species and also describing ages of nestlings

^3^Articles outlining growth and survivorship of nestlings as they relate to age

^4^Articles comparing development between species

**Appendix References**

1. Jongsomjit D, Jones SL, Geupel GR, Grouse P (2007) A guide to nestling development and aging in altricial passerines. US Fish and Wildlife Service Biological Technical Publication FWS/BTP-R6008-2007.
2. Rodway MS (1997) Relationship between wing length and body mass in Atlantic Puffin chicks. J Field Ornithol 68: 338-347.
3. Morales Fernaz J, Schifferli L, Grüebler MU (2012) Ageing nestling Barn Swallows *Hirundo rustica*: An illustrated guide and cautionary comments. Ringing Migr 72: 65-75.
4. Coleman JS, Fraser JD (1989) Age estimation and growth of Black and Turkey Vultures. J Field Ornithol 60: 197-208.
5. Custer TW, Peterson DW Jr (1991) Growth rates of Great Egret, Snowy Egret and Black-crowned Night-heron chicks. Waterbirds 14: 46-50.
6. Dane CW (1968) Age determination of Blue-winged Teal. J Wildl Manage 32: 267-274.
7. Mañosa S, Codina J (1995) Age estimation and growth patterns in nestling Bonelli's Eagles. J Raptor Res 29: 273-275.
8. Lyons DM, Mosher JA (1983) Age-estimation model for nestling Broad-winged Hawks. Wildl Soc Bull 11: 268-270.
9. Smith JE, Diem KL (1972) Growth and development of young California Gulls (*Larus californicus*). Condor 74: 462-470.
10. Yocom CF, Harris SW (1965) Plumage descriptions and age data for Canada Goose goslings. J Wildl Manage 29: 874-877.
11. Dzubin A (1959) Growth and plumage development of wild-trapped juvenile Canvasback (*Aythya valisineria*). J Wildl Manage 23: 279-290.
12. Hamel PB (1974) Age and sex determination of nestling Common Grackles. Bird Banding 45: 16-23.
13. Wagener M (1998) Praktische Hinweise für brutbiologische Untersuchungen an der Flußseeschwalbe *Sterna hirundo*. Vogelwelt 119: 279-286.
14. Penteriani V, Delgado MDM, Maggio C, Aradis A, Sergio F (2005) Development of chicks and predispersal behaviour of young in the Eagle Owl *Bubo bubo*. Ibis 147: 155-168.
15. Murphy MT (1981) Growth and aging of nestling Eastern Kingbirds and Eastern Phoebes. J Field Ornithol 52: 309-316.
16. Moritsch MQ (1985) Photographic guide for aging nestling Ferruginous Hawks. Boise, ID: USDI Bureau Land Management.
17. Poole KG (1989) Determining age and sex of nestling Gyrfalcons. J Raptor Res 23: 45-47.
18. Hunter JM (1995) A key to ageing goslings of the Hawaiian Goose *Branta sandvicensis*. Wildfowl 46: 55-58.
19. Reed JM, Gray EM, Lewis D, Oring LW, Coleman R, et al. (1999) Growth patterns of Hawaiian Stilt chicks. Wilson Bull 111: 478-487.
20. Elowe KD, Payne S (1979) Aging young Herring Gulls from measurements of body parts. Bird Banding 50: 49-55.
21. Mineau P, Smith GJ, Markel R, Lam CS (1982) Aging Herring Gulls from hatching to fledging. J Field Ornithol 53: 394-402.
22. Ramanujam ME, Murugavel T (2009) A preliminary report on the development of young Indian Eagle Owl *Bubo bengalensis* in and around Puducherry, southern India. J Threat Taxa 1: 519-524.
23. Hailman JP (1961) Age of Laughing Gull chicks indicated by tarsal length. Bird Banding 32: 223-226.
24. Reed LM, Caccamise DE, Orrell EP (1998) Aging Laughing Gull nestlings using head-bill length. Waterbirds 21: 414-417.
25. Horwich RH (1966) Feather development as a means of aging young Mockingbirds (*Mimus polyglottos*). Bird Banding 37: 257-267.
26. Saunders MB, Hansen GL (1989) A method for estimating the ages of nestling Northern Harriers (*Circus cyaneus*). Can J Zool 67: 1824-1827.
27. Moritsch MQ (1983) Photographic guide for aging nestling Prairie Falcons. Boise, ID: USDI Bureau Land Management.
28. Podlesak DW, Blem CR (2002) Determination of age of nestling Prothonotary Warblers. J Field Ornithol 73: 33-37.
29. Parr R (1975) Aging Red Grouse chicks by primary molt and development. J Wildl Manage 39: 188-190.
30. Penak BL, Dykstra CR, Miller SJ, Bird DM (2013) Using morphometric measurements to estimate age of nestling Red-shouldered Hawks in two eastern populations. Wilson J Ornithol 125: 630-637.
31. Moritsch MQ (1983) Photographic guide for aging nestling Red-tailed Hawks. Boise, ID: USDI Bureau Land Management.
32. Holcomb LC, Twiest G (1971) Growth and calculation of age for Red-winged Blackbird nestlings. Bird Banding 42: 1-17.
33. Lesage L, Reed A, Savard JPL (1996) Plumage development and growth of wild Surf Scoter *Melanitta perspicillata* ducklings. Wildfowl 47: 199-203.
34. Eriksson D, Lundberg A, Westman B (1984) Estimating age of Ural Owl nestlings from body part measurements. Ann Zool Fennici 21: 313-316.
35. Fautin RW (1941) Development of nestling Yellow-headed Blackbirds. Auk 58: 215-232.
36. Weaver RL (1942) Growth and development of English Sparrows. Wilson Bull 54: 183-191.
37. Hoyt JSY (1944) Preliminary notes on the development of nestling Pileated Woodpeckers. Auk 61: 376-384.
38. Bateman GC, Balda RP (1973) Growth, development, and food habits of young Pinyon Jays. Auk 90: 39-61.
39. Beason RC, Franks EC (1973) Development of young Horned Larks. Auk 90: 359-363.
40. Dunn EH (1975) Growth, body components and energy content of nestling Double-crested Cormorants. Condor 77: 431-438.
41. Hadow HH (1976) Growth and development of nestling Downy Woodpeckers. North American Bird Bander 1: 155-164.
42. Clay DL, Brisbin IL Jr, Youngstrom KA (1979) Age-specific changes in the major body components and caloric values of growing Wood Ducks. Auk 96: 296-305.
43. Moss D (1979) Growth of nestling Sparrowhawks (*Accipiter nisus*). J Zool 187: 297-314.
44. Threlfall W, Cannings RJ (1979) Growth of nestling Savannah Sparrows. Bird Banding 50: 164-166.
45. Brown PW, Fredrickson LH (1983) Growth and moult progression of White-winged Scoter ducklings. Wildfowl 34: 115-119.
46. Sedinger JS (1986) Growth and development of Canada Goose goslings. Condor 88: 169-180.
47. Holland PK, Yalden DW (1991) Growth of Common Sandpiper chicks. Wader Study 69: 114-117.
48. Magrath RD (1991) Nestling weight and juvenile survival in the Blackbird *Turdus merula*. J Anim Ecol 60: 335-351.
49. Gilliland SG, Ankney CD (1992) Estimating age of young birds with a multivariate measure of body size. Auk 109: 444-450.
50. Nagarajan R, Thiyagesan K, Natarajan R, Kanakasabai R (2002) Patterns of growth in nestling Indian Barn-Owls. Condor 104: 885-890.
51. Seidensticker MT, Flockhart DTT, Holt DW, Gray K (2006) Growth and plumage development of nestling Long-eared Owls. Condor 108: 981-985.
52. Banks RC (1959) Development of nestling White-crowned Sparrows in central coastal California. Condor 61: 96-109.
53. Scharf WC, Balfour E (1971) Growth and development of nestling Hen Harriers. Ibis 113: 323-329.
54. Barrett RT, Runde OJ (1980) Growth and survival of nestling Kittiwakes *Rissa tridactyla* in Norway. Ornis Scand 11: 228-235.
55. Dunn EH, Brisbin L Jr (1980) Age-specific changes in the major body components and caloric values of Herring Gull chicks. Condor 82: 398-401.
56. Winterstein SR, Raitt RJ (1983) Nestling growth and development and the breeding ecology of the Beechey Jay. Wilson Bull 95: 256-268.
57. Haggerty TM (1994) Nestling growth and development in Bachman’s Sparrows. J Field Ornithol 65: 224-231.
58. Lesage L, Gauthier G (1997) Growth and development in Greater Snow Goose goslings. Auk 114: 229-241.
59. McCarty JP, Winkler DW (1999) Relative importance of environmental variables in determining the growth of nestling Tree Swallows *Tachycineta bicolor*. Ibis 141: 286-296.
60. Blais S, Guillemain M, Durant D, Fritz H, Guillon N (2001) Growth and plumage development of Pintail ducklings. Wildfowl 52: 69-86.
61. Naef-Daenzer B, Keller LF (1999) The foraging performance of Great and Blue Tits (*Parus major* and *P. caeruleus*) in relation to catepillar development, and its consequences for nestling growth and fledging weight. J Anim Ecol 68: 708-718.
62. Becker PH, Wink M (2003) Influences of sex, sex composition of brood, and hatching order on mass growth in Common Terns *Sterna hirundo*. Behav Ecol Sociobiol 54: 136-146.
63. Benowitz-Fredericks ZM, Kitaysky AS, Thompson CW (2006) Growth and allocation in captive Common Murre (*Uria aalge*) chicks. Auk 123: 722-734.
64. Pande S, Pawashe A, Mahajan MN, Mahabal A, Yosef R, et al. (2011) Biometry based ageing of nestling Indian Spotted Owlets (*Athene brama brama*). ZooKeys 132: 75-88.
65. Kear J (1970) Studies on the development of young Tufted Duck. Wildfowl 21: 123-132.
66. Balph MH (1975) Development of young Brewer's blackbirds. Wilson Bull 87: 207-230.
67. Pinkowski BC (1975) Growth and development of Eastern Bluebirds. Bird Banding 46: 273-289.
68. Ricklefs RE, White SC (1981) Growth and energetics of chicks of the Sooty Tern (*Sterna fuscata*) and Common Tern (*S. hirundo*). Auk 98: 361-378.
