# Supplementary material for: Are Morphometrics Sufficient for Estimating Age of Pre-Fledging Birds in the Field? A Test Using Common Terns (Sterna hirundo)
Source: PLoS One. 2014 Nov 6;9(11):e111987. doi: 10.1371/journal.pone.0111987 (PMC4222966; doi:10.1371/journal.pone.0111987)
Supplement: Table S2 — Bias in estimation errors during all tests of the tool. (DOCX) [file pone.0111987.s003.docx]

**Table S2.** Bias in estimation errors during all tests of the tool. Percent of chick age estimations that were either under-estimated by one age group, over-estimated by one age group, correctly estimated actual age group, or were completely incorrect for each age group. Percentages calculated are for each corresponding age group. Observers experienced the most difficulty estimating chick ages for chicks in Groups 4 and 5 (13 – 19 days), both with and without the tool and underestimated age for Groups 5 and 6 (20 – 23 days) more when using the tool.

| *Without* | | | | | |
| --- | --- | --- | --- | --- | --- |
| Group | Under-Estimate | Actual Age | Over-Estimate | Incorrect | n |
| 1 | --- | 64.5% | 28.9% | 6.6% | 16 |
| 2 | 24.1% | 38.4% | 26.8% | 10.8% | 18 |
| 3 | 27.3% | 39.5% | 25.9% | 7.2% | 29 |
| 4 | 36.1% | 26.9% | 18.6% | 18.4% | 29 |
| 5 | 24.7% | 32.5% | 25.3% | 17.5% | 33 |
| 6 | 24.0% | 71.3% | --- | 4.7% | 6 |
| *With* | | | | | |
| Group | Under-Estimate | Actual Age | Over-Estimate | Incorrect | n |
| 1 | --- | 93.0% | 6.6% | 0.3% | 12 |
| 2 | 36.5% | 60.0% | 3.2% | 0.3% | 20 |
| 3 | 28.9% | 66.4% | 3.4% | 1.2% | 29 |
| 4 | 36.1% | 51.0% | 6.7% | 6.2% | 27 |
| 5 | 50.9% | 38.8% | 4.7% | 5.6% | 32 |
| 6 | 42.3% | 50.4% | --- | 7.4% | 6 |
