# Supplementary material for: Are Morphometrics Sufficient for Estimating Age of Pre-Fledging Birds in the Field? A Test Using Common Terns (Sterna hirundo)
Source: PLoS One. 2014 Nov 6;9(11):e111987. doi: 10.1371/journal.pone.0111987 (PMC4222966; doi:10.1371/journal.pone.0111987)
Supplement: Table S3 — Model averaging results for learning-phase tests. (DOCX) [file pone.0111987.s004.docx]

**Table S3.** Model averaged components, standard errors, confidence intervals, and relative importance of predictors across all possible GLMMs predicting observer chick-aging accuracy in learning-phase trials. Predictors are shown with levels in [] and back-transformed means are provided for main effects. Predictors are ranked by their relative importance (the sum of all Akaike weights for each of the models in which the predictor was included); “:” indicates an interaction term.

| Predictor [Level] | Model averaged means | Model averaged component | Component SE | Lower 95% CI | Upper 95% CI | Relative importance |
| --- | --- | --- | --- | --- | --- | --- |
| Chick age [Group 2] | 0.40 | -0.41 | 0.22 | -0.76 | -0.05 | 0.98 |
| Chick age [Group 3] | 0.58 | 0.33 | 0.34 | -0.22 | 0.89 |  |
| Chick age [Group 4] | 0.46 | -0.17 | 0.31 | -0.68 | 0.34 |  |
| Chick age [Group 5] | 0.54 | 0.16 | 0.34 | -0.40 | 0.72 |  |
| Chick age [Group 6] | 0.91 | 2.32 | 0.81 | 0.98 | 3.65 |  |
| Experience [Some] | 0.33 | -0.69 | 0.30 | -1.19 | -0.20 | 0.67 |
| Experience [Most] | 0.41 | -0.35 | 0.32 | -0.88 | 0.17 |  |
| Location [Little Island, NY] | 0.46 | -0.16 | 0.17 | -0.44 | 0.11 | 0.64 |
| Location [Presqu'ile, ON] | 0.55 | 0.21 | 0.35 | -0.37 | 0.79 |  |
| Tool use [without tool] | 0.36 | -0.55 | 0.25 | -0.97 | -0.14 | 0.62 |
| Tool use [with tool] | 0.53 | 0.10 | 0.34 | -0.46 | 0.66 |  |
| Experience [Most] : Location [Little Island, NY] |  | -1.71 | 0.67 | -2.81 | -0.61 | 0.50 |
| # of previous trials [0] | 0.42 | -0.32 | 0.17 | -0.60 | -0.04 | 0.39 |
| # of previous trials [1] | 0.51 | 0.04 | 0.24 | -0.35 | 0.44 |  |
| Experience [Most] : Tool use [with tool] |  | 0.25 | 0.41 | -0.42 | 0.92 | 0.13 |
| Experience [Most] : # of previous trials [1] |  | 0.20 | 0.41 | -0.48 | 0.88 | 0.08 |
| Tool use [with tool] : # of previous trials [1] |  | 0.29 | 0.40 | -0.36 | 0.94 | 0.08 |
| Experience [Most] : Chick age [Group 3] |  | 0.37 | 0.62 | -0.64 | 1.38 | 0.03 |
| Experience [Most] : Chick age [Group 4] |  | -0.22 | 0.59 | -1.20 | 0.76 |  |
| Experience [Most] : Chick age [Group 5] |  | 0.62 | 0.60 | -0.36 | 1.60 |  |
| Experience [Most] : Chick age [Group 6] |  | -0.23 | 1.57 | -2.82 | 2.36 |  |
| Experience [Most] : Location [Little Island, NY : # of previous trials [1] |  | 0.03 | 0.80 | -1.30 | 1.35 | <0.01 |
